# *TP53*,* SPOP* and *PIK3CA* Genes Status in Prostate Cancer

**DOI:** 10.31557/APJCP.2020.21.11.3365

**Published:** 2020-11

**Authors:** Mazhar Salim Al Zoubi, Raed Otoum, Mohammed S Alorjani, Samir Al Bashir, Bahaa Al Trad, Manal Issam Abualrja, Sohaib M Al-Khatib, Khalid Al-Batayneh

**Affiliations:** 1 *Department of Basic Medical Sciences, Faculty of Medicine, Yarmouk University, Irbid 211-63, Jordan. *; 2 *Department of Biological Sciences, Faculty of Science, Yarmouk University, Irbid 211-63, Jordan. *; 3 *Departments of Pathology and Microbiology, Faculty of Medicine, Jordan University of Science and Technology, Irbid, Jordan.*

**Keywords:** TP53, SPOP, PIK3CA, prostate cancer, p53

## Abstract

Recent advances in molecular biology make the identification of prostate cancer (PC) subsets a priority for more understanding of the molecular pathogenesis and treatment options. Genetic alterations in many genes such as *TP53*, *SPOP* and *PIK3CA* genes have been reported in PC with variable frequencies worldwide. We aimed to investigate genetic alterations in the hotspot lesions of *TP53*, *SPOP* and *PIK3CA* genes by direct sequencing and the expression of *TP53* and *PIK3CA* by RT-PCR in prostate cancer, and to explore the correlation between *TP53*, *SPOP* and *PIK3CA* alterations and tumorigenesis of prostate cancer. Seventy-nine FFPE prostate samples from patients who underwent radical prostatectomy were obtained, subjected to genomic DNA extraction and sequenced for mutations in exons 5, 6, 7 and 8 of *TP53* gene, exons 4 and 5 of *SPOP* gene and exons 9 and 20 of *PIK3CA* gene. RT-PCR was performed for the expression evaluation of the *PIK3CA* gene. Our results showed a high frequency of *TP53* mutations (11/79, 13.9 %) in the selected population. On the other hand, *SPOP* and *PIK3CA* genes did not show any genetic alteration in the sequenced exons. *PIK3CA* gene overexpression was detected in 6% of the cohort by RT-PCR. *TP53* mutation is the most frequent genetic alteration and likely has a major role in the pathogenesis of PC in the Jordanian population.

## Introduction

Prostate cancer (PC) is considered the second most common type and the second leading cause of deaths of male cancers and the fourth common type of cancer worldwide (Ferlay et al., 2015; Siegel et al., 2017; Siegel et al., 2019). In Jordan, PC has been estimated to be the fourth most common cancer in males (8.4 %) and the third cause of death with a 6.2 % mortality rate among males (Abdel-Razeq et al., 2015). Age, family history, race, lifestyle, diet, environmental and genetic factors have been proposed as PC risk factors (Ballon-Landa and Parsons, 2018). To build a clear portrait of PC, intensive molecular investigations are conducted to generate a distinctive molecular profile of PC, however, the molecular pathogenesis of PC is not fully understood (Abeshouse et al., 2015). Voluminous genetic alterations have been proposed and studied to understand the etiology and molecular pathology of prostate cancer. For instance, *TP53*, *SPOP*, *PIK3CA*, BRCA2 and AR genes alterations have been demonstrated in PC in various populations (Bookstein et al., 1993; Carroll et al., 1993; Taplin et al., 1995; Li et al., 1997; Edwards et al., 2003c; Thomas et al., 2007; Taylor et al., 2010; Barbieri et al., 2012; Beltran et al., 2013). Despite the presence of different patterns of genetic alterations in these hotspot genes, the fallouts of these studies did not nominate a specific gene alteration in association with the development of PC since different studies showed a variable prevalence of *TP53*, *SPOP* and *PIK3CA* mutations or alterations in the PC cases (Bookstein et al., 1993; Carroll et al., 1993; Mirchandani et al., 1995; Edwards et al., 2003b; Müller et al., 2007; Sun et al., 2009; Taylor et al., 2010; Barbieri et al., 2012; Abeshouse et al., 2015). The presence of the *TP53* mutation in PC cell lines shed a light on the impact of the *TP53* alterations in the development of PC (Carroll et al., 1993). Further studies showed variable frequencies of *TP53* mutations in PC cases (Bookstein et al., 1993; Voeller et al., 1994; Mirchandani et al., 1995; Thomas et al., 2007; Barbieri et al., 2012; Abeshouse et al., 2015). As well, mutations in the *SPOP* (Speckle-type POZ protein) gene have been also detected in PC cases with variable prevalence (Barbieri et al., 2012; Blattner et al., 2014; Abeshouse et al., 2015). Besides, the *PIK3CA* gene alteration is highly prevalent in many types of cancers such as endometrial, breast, ovarian, colorectal and prostate cancers (Campbell et al., 2004; Samuels et al., 2004; Oda et al., 2005; Taylor et al., 2010). Therefore, identification of molecular idiosyncratic subsets of PC is the ultimate goal for more understanding the molecular pathology and treatment regimen. 

Consequently, the present study was conducted to investigate the role of genetic alterations in *TP53*, *SPOP* and *PIK3CA* genes in prostate cancer of a Jordanian cohort. We aimed to perform direct sequencing for the hotspot mutations in exons 5, 6, 7 and 8 of the *TP53* gene, exons 9 and 20 of *PIK3CA* gene and exons 4 and 5 of *SPOP* gene. Moreover, the expression level of *PIK3CA* gene was assessed by RT-PCR.

## Materials and Methods

*Tumor Samples and Patients*

Sample collection and acquiring data were performed after obtaining the institutional review board (IRB) approval from King Abdullah University Hospital (KAUH) at Jordan University of Science and Technology, Irbid (IRB #: 13/1/881 and Hospital Policy: GM7601). 

Formalin-Fixed Paraffin-Embedded (FFPE) tissue samples from 79 PC patients who underwent prostatectomy were provided by the Department of Pathology at KAUH from January 2003 through December 2015. The tissue diagnoses were submitted based on a pathologic assessment of physicians who requested the assays and were further verified by a pathologist at the Department of Pathology. We specifically chose samples from the primary tumor of each original specimen where cancer cells were identified. Macrodissection was performed only on a single representative block from the primary tumor. The majority of the samples showed tumor component of over 60%. The mean age of the enrolled patients with PC was 72 years (55–95 years).

*DNA Extraction*

Genomic DNA was extracted from (FFPE) by using the QIAmp extraction DNA kit (Qiagen GmbH, Hilden, Germany) according to the manufacturer’s protocols. Briefly, five FFPE sections were collected in 1.5 mL Eppendorf tubes. Deparaffinization and clearing process was conducted using xylene (3 times) and ethanol incubation (2 times), spinning and drying. The last precipitate was incubated with the lysis buffer, incubated for two hours, followed by column wash and finally eluted with elution buffer. All eluents were stored in -80^o^C until use. The purity of DNA was estimated by using Nano-drop and confirmed by 0.5 % agarose gel electrophoresis and DNA samples were stored at -20^o^C for long time storage for further analysis.

*Polymerase Chain Reaction (PCR)*

The PCR amplifications targeting exons 5, 6, 7 and 8 of *TP53* gene, exons 4 and 5 of *SPOP* gene and exons 9 and 20 of *PIK3CA* gene were performed using specific sets of primers for the detection of any point mutations based on the NCBI gene sequences (Table 1). 

The PCR amplification reaction was performed in a total reaction volume of 30 μL using 2X ready to use a master mix from (New England BioLabs, USA). The PCR reaction was conducted in Gene Pro thermal cycler model TC-E-96G (Bioer, China) under the following cycling conditions: Initial denaturation at 94^o^C for 3 minutes; 40 cycles of 94^o^C denaturation for 30 seconds, Tm^o^C annealing for 30 seconds, 68^o^C extension for 60 seconds; followed by final extension at 68^o^C for 5 minutes. PCR products were analyzed and resolved by running the samples on 1.5 % agarose gel. 

*DNA Sequencing*

Sanger DNA sequencing was performed by external service by (GENEWIZ, NJ, USA). The output of the DNA sequencing service was analyzed by Mutation Surveyor V5.1.1 and FinchTV 1.5. When the sample shows a mutation it was repeated in duplicate in a separate run by performing a PCR and Sanger sequencing. 

*RNA Extraction, cDNA synthesis and qRT-PCR*

RNA extraction was performed by total RNA purification kit from (Jena Bioscience, Germany). Then, the cDNA was produced by following the manufacturer’s instructions using RevertAid First Strand cDNA Synthesis Kit from (Thermo Scientific, Lithuania). Finally, the qRT-PCR was conducted using Maxima SYBR Green qPCR Master Mix (2X) kit from (Thermo Scientific), while the amplification was performed on LineGene 9600 Plus thermal cycler from (Bioer, China). The qRT-PCR analysis was performed by using 2^-∆Ct^ calculation relative to the β-actin reference gene using the following primers (PIK3CA: F-5’- GGCCACTGTGGTTGAATTGGG -3’, R-5’- AGTGCACCTTTCAAGCCGCC -3’), (β-actin: F-5’- GAA CGG TGA AGG TGA CAG -3’, R-5’- TTT AGG ATG GCA AGG GAC T -3’). 

## Results

Seventy-nine PC samples were screened for mutations in exons 5, 6, 7 and 8 of *TP53* gene (NG_017013) (HGNC: 11998), exons 4 and 5 of *SPOP* (NG_041815) (HGNC:11254) gene and exons 9 and 20 of PIK3CA (NG_012113) (HGNC:8975) gene. The clinicopathological data of patients are represented in Table 2.

**Figure 1:**
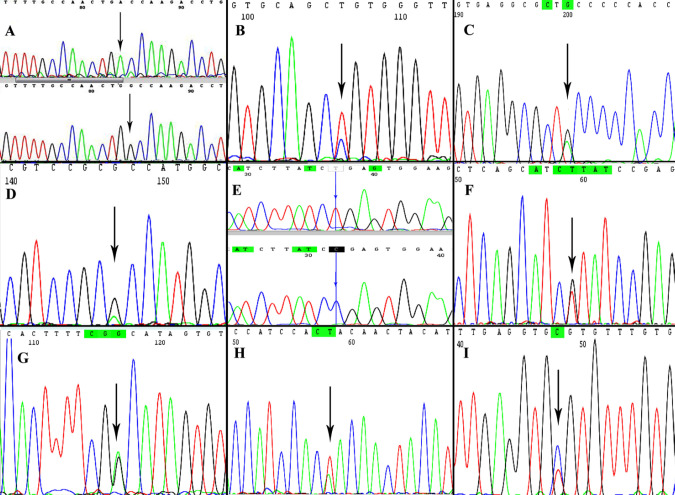
A Representative Chromatogram of the Detected Mutations in the *TP53* Gene. A: Homologous mutation GCC/ACC (A1358T) (exon 5), B: CTG/CCG (L145P) (exon 5), C: TGC/TAC (C176Y) (exon 5), D: Rs868157297 (G/GA) (intron 4), E: Homologous mutation CGA/TGA (R196*) (exon 6), F: CTT/CGT (L194R) (exon 6), G: CGA/CGG (R213R) (exon 6) H: TAC/AAC (Y234N) (exon 7), I: CGT/TGT (R273C) (exon 8)

The current results showed a substantial frequency of mutations in the *TP53* gene (13.9 %, 11/79) as shown in Table 3. Missense mutations in the *TP53* gene were detected in nine samples as the followings: four substitutional mutations were detected in exon 5 (A138T, L145P, 2 C176Y) and one substitutional mutation in intron 5 (C12351T), one substitutional mutation was detected in exon 6 (L194R), two substitutional mutations were detected in exon 7 (Y234N), and one substitutional mutation was detected in exon 8 (R273C). In addition, a termination mutation was detected in exon 6 (R196X). Finally, a silent mutation was detected in exon 6 (R213R). A sample of detected mutations is presented in Figure 1. The detailed description of the detected mutations is illustrated in Table 3.

On the other hand, our findings did not show any mutation in exons 4 and 5 of *SPOP* gene and exons 9 and 20 of *PIK3CA* gene. Also, the RT-PCR analysis showed overexpression in 6% of the *PIK3CA* gene in the selected samples.

**Table 1:** The Set of Primers Used to Detect the Point Mutations on Exon 5, 6, 7 and 8 of the *TP53* Gene, Exon 4 and 5 of SPOP Gene, and Exon 9 and 20 of *PIK3CA* Gene

Target Gene	Exon	Forward primer	Reverse primer	Tm (^o^C)
*TP53*	5	F- 5’CAC TTG TGC CCT GAC TTT CAA C-3’	R- 5’- CAA CCA GCC CTG TCG TCT CTC -3’	56
	6	F- 5’-TCC CCA GGC CTC TGA TTC CT-3’	R- 5’- CCT TAA CCC CTC CTC CCA GA -3’	56
	7	F- 5’- GCC TCA TCTTGG GCC TGTGTTATC-3’	R- 5’- TCA GAG GCA AGC AGA GGC TG -3’	56
	8	F- 5’-CTGATTTCCTTACTGCCTCTTGC -3’	R- 5’- TCTCCTCCACCGCTTCTTGTC -3’	56
*SPOP*	4	F-5'-ACCCATAGCTTTGGTTTCTTCTCCC-3'	R-5'-TATCTGTTTTGGACAGGTGTTTGCG-3'	60
	5	F-5'-ACTCATCAGATCTGGGAACTGC-3'	R-5'-AGTTGTGGCTTTGATCTGGTT-3'	60
*PIK3CA*	9	5'-CTGTGAATCCAGAGGGGAAA-'3	5'-CCACAAATATCAATTTACAACCATTG-'3	55
	9	5'-AATCCAGAGGGGAAAAATATGA-'3	5'-TGAGATCAGCCAAATTCAGTTA-'3	55
	20	5'-GCTTTGTCTACGAAAGCCTCTCT-'3	5'-ATACATTCGAAAGACCCTAGCCC-'3	60
	20	5'-ATGATGCTTGGCTCTGGAAT-'3	5'-ACTCTCAGCAGGCAAAGACC-'3	60

**Table 2:** Clinicopathological Data of the Patients

Clinicopathological data (n = 79)		
Age (years)	72	
PSA (ug/L)	61.6	
Gleason Score	n	%
3+3	10	13%
3+4	20	25%
4+3	3	4%
4+4	10	13%
4+5	21	26%
5+4	3	4%
5+5	12	15%

**Table 3:** Mutation Frequency in the *TP53* Gene in the Prostate Samples

Gene	Exon #	Point mutation	bp substitution	Genomic Mutation ID	Mutation (CDS)	Common in
*TP53*	5	A138T	GCC/ACC (12400)	COSV52839281	c.412G>A	Breast Cancer (Maitra et al., 1999), Oral Squamous Carcinoma(Sakata, 1996)
		L145P	CTG/CCG (12422)	COSV52676080	c.434T>C	Colorectal Cancer(Yuen et al., 1997), Breast Cancer (Umekita et al., 1994), Ovarian Cancer (Fallows et al., 2001)
		2 C176Y	TGC/TAC (12515)	COSV52660760	c.527G>A	Colorectal Cancer(Onda et al., 1997; Yuen et al., 1997), Lung Cancer (Husgafvel-Pursiainen et al., 2000) Breast Cancer (Falette et al., 1998)
		C/CT	C/CT	rs868137297	C/CT	Anaplastic rhabdomyosarcoma, (Hettmer et al., 2014) Li-Fraumeni syndrome
			-12351	NM_000546.5(TP53):c.376-1G>A AND Ovarian Neoplasms	NG_017013.2:g.17314G>A	(Villani et al., 2016) and (Li et al., 2019), Breast cancer (Li et al., 2019).
	6	L194R	CTT/CGT (12650)	COSV52679257	c.581T>G	Breast(Andersen et al., 1993) and lung(Suzuki et al., 1992)
		R196*	CGA homo TGA (12655)	COSV52663748	c.586C>T (Homologus)	Gastric Cancer(Poremba et al., 1995), breast (Chappuis et al., 1999)Esophagus(Audrezet et al., 1993), Skin Cancer (Moles et al., 1993)
		R213R	CGA/CGG (12708)		c.639A>G	CNS (Ohgaki et al., 1993) Biliary tract (Wardell et al., 2018), Colorectal Cancer (Ashktorab et al., 2017)
	7	2 Y234N	TAC/AAC (13337)	COSV52730114	c.700T>A	Prostate cancer(Konishi et al., 1995), B-cell lymphoma (Zhang et al., 2013), and breast cancer(Van Emburgh et al., 2008)
	8	R273C	CGT/TGT (13797)	COSV52662066	c.817C>T	Colorectal cancer(Ishioka et al., 1991), hepatocellular carcinoma (Kress et al., 1992) , Esophageal carcinoma (İmazeki et al., 1992)

## Discussion

Structural and functional alterations in an immense number of genes have been found in PC tissues involving germline, somatic and mitochondrial genetic alterations (Dong, 2006). However, due to variable prevalence in these mutations in PC, there is a need for more genetic studies to identify the molecular portrait of PC. Therefore, we aimed to investigate genetic alteration in the most predominant genetically mutated genes; *TP53*, *SPOP* and *PIK3CA* in most cancers. 

The p53 transcription factor has a central role in the induction of cell suicide process in response to DNA damage or oncogenesis activation (Sherr and McCormick, 2002). Therefore, alterations in the *TP53* gene are expected to be associated with the development of several cancers, including PC. Our results showed a distinctive pattern of genetic alteration in the Jordanian PC which was demonstrated by the presence of *TP53* mutation in 13.9 % of the studied population. The detected *TP53* mutations were found in exons 5, 6, 7 and 8. Genetic alterations in *TP53*, as a tumor suppressor oncogene, have been reported as a predominant mutated gene in many cancers (Bookstein et al., 1993; Carroll et al., 1993; Mirchandani et al., 1995; Sherr and McCormick, 2002; Dong, 2006; Kandoth et al., 2013). In 1992, Effert et al, reported the presence of point mutation in codon 172 of *TP53* gene (Effert et al., 1992). Then, propagation in *TP53* genetic studies showed a presence of various prevalence of *TP53* mutations in different populations ranging from 2 to 65 % (Hall et al., 1995; Hughes et al., 1995; Kubota et al., 1995; Gumerlock et al., 1997; Theodorescu et al., 1997; Kluth et al., 2014). Moreover, some studies showed an association between *TP53* mutations and the advanced stages of PC (Bookstein et al., 1993; Abate-Shen and Shen, 2000). While other studies demonstrated a frequently expressed transition mutations (Chi et al., 1994). Underscoring the importance of p53 pathway, a mutation in CHEK2, an upstream regulator of p53, has also been found to be frequent in PC development (Dong et al., 2003).

Oncogenic mutations in *TP53* have been shown in different cancer cell lines which may involve invasion, migration, propagation of cell cycle, drug resistance, anchorage-independent growth, increased colony formation, polyploidy and angiogenesis (Effert et al., 1992; Muller and Vousden, 2014). The presence of *TP53* mutation pattern in PC can be a worthy diagnostic tool for the identification of a subset of patients that may undergo a treatment regimen since some studies showed a role of mutant *TP53* in the signaling pathways such as RTK (Adorno et al., 2009; Sauer et al., 2010; Wang et al., 2013).

Here, we identified eleven point-mutations in the DNA binding domain of the *TP53* gene that has been reported in previous studies. Specifically, A138T mutation was reported in lung and gastric cancers (Bumroongkit et al., 2008; Shimizu et al., 2014), L145P mutation was reported in breast cancer (Greenblatt et al., 2001), C176Y mutation was reported in ovarian cancer, L194R was reported in lung cancer (Ko et al., 2002), R196* was described as a truncated mutation the produces a pro-tumorigenic isoforms and reported in colon cancer (Shirole et al., 2016), Y234N was associated with prostate cancer (Konishi et al., 1995) and breast cancer (Van Emburgh et al., 2008), R273C and gastric cancer(Renault et al., 1993) and hepatocellular carcinoma (Kress et al., 1992). 

It is believed that *TP53* mutations are associated with the metastasis and progression of PC (Eastham et al., 1995; Dong, 2006; Ecke et al., 2010). As a limitation of our study, the clinical data of the patients were not available for the follow-up because most of them were referred to the central cancer centre (King Hussain Cancer Center, Amman). However, other genetic alterations in the *TP53* gene for the same population are highly recommended to be investigated by exome sequencing to generate a clear molecular portrait of the *TP53* gene in the Jordanian PC patients. 

On the other hand, *SPOP* and *PIK3CA* genes did not show any mutation or a variation in the gene expression at the level of mRNA. *SPOP* gene mutation has been reported as one of the most frequents alterations in PC (Barbieri et al., 2012; Abeshouse et al., 2015). Abeshouse et al, in a molecular taxonomy study, have reported a mutation in the *SPOP* gene in 11 % of primary PC samples (Abeshouse et al., 2015). A diverse population cohorts showed variable prevalence of *SPOP* mutations in PC (4.6 % - 14.4 %) (Blattner et al., 2014). The whole-exome sequencing of multiple cohorts showed *SPOP* mutations in 6-15 % of primary and metastatic PC (Barbieri et al., 2012). Another study conferred the role of mutated *SPOP* gene in the resistance of PC treatment (Dai et al., 2017). Furthermore, Gunther et al demonstrated the role of *SPOP* mutations in the genomic instability in PC (Boysen et al., 2015). The inconsistency of our findings with the previous reports can be attributed to the different exons that have been studied. Therefore, it is recommended to screen the rest exons in the same population. Our findings did not exclude the possible role of *SPOP* in the development of PC since we sequenced only two exons (4 and 5) of the *SPOP* gene. Therefore, further genetic studies by exome sequencing are required to investigate the prevalence of *SPOP* mutations in PC of the Jordanian population. 

*PIK3CA* oncogenic alteration in many cancers derived the hypothesis of its role in the development of PC. Genomic alterations of the PI3K-AKT signaling pathway have been reported in many diverse solid tumors including endometrial, colorectal, cervical, Salivary gland, breast, gastric, ovarian and prostate cancers (Bachman et al., 2004; Samuels et al., 2004; Levine et al., 2005; Sun et al., 2009; Arsenic et al., 2014; Millis et al., 2016). Three studies have reported *PIK3CA* alterations in PC (Edwards et al., 2003a; Müller et al., 2007; Sun et al., 2009). Two of them described *PIK3CA* amplification and only the most recent study by Sun et al., (2009) has found for the first time *PIK3CA* mutations in exon 9 in one sample representing 3% of prostate tumors. Consistently, our findings support the rare incidence of *PIK3CA* mutations and overexpression in a subset of PC cases (6%) compared to a previous study that reported about 13% of the cases (Sun et al., 2009). The oncogenic influence can be stimulated through gene amplification, point mutation, and translocation (Nathanson et al., 2001). A point mutation may not be the main pathway to activate *PIK3CA* gene in PC in this study. PI3Ks signaling pathway also has many other components besides *PIK3CA* gene which ultimately results in the activation of PI3K-AKT signaling pathway. The current data, together with other studies are not demonstrating the involvement of *PIK3CA* mutation in PC, however, other components of the PI3K pathway in these tumors may be involved in PC tumorigenesis and suggest that the PI3K pathway may exhibit a valuable target for the development of novel therapies for this type of cancer. 

Tumor growth and development is a multifactorial disorder, which requires a multistep process involving many cell processes that include signal transduction, cell metabolism and cell cycling. One of the most important mechanisms that in turn plays a role in activating oncogenes or inactivating tumor suppressor genes is gene aberration. Jordanian differences in genetic makeup, smoking consumption, diet habits, and other unidentified cultural factors may also be responsible for the disparity. The diverse pathological and etiological factors of PCs in different geographical locations may be paralleled by differences in the molecular pathway of tumor development. 

The lack of mutations in *PIK3CA* in the current cohort of PC articulates that mutations in this gene are not a key element in PC pathogenesis in the Jordanian population. This reveals that *PIK3CA* gene mutation is not the primary molecular mechanism in activating the PIK3/AKT-driven tumorigenesis pathway in PC. These results show a quite good congruence with previously published studies, most of them based on a smaller number of cases or not precisely defining the clinicopathological features of the samples but in our study, we have selected a larger, well-defined group of patients.

Since the current study is only constricted on a radical prostatectomy sample from different Gleason scores, further studies involving PC biopsies from different patients may be able to address the *TP53*, *SPOP* and *PIK3CA* mutations in PC. Our study was biased only for radical prostatectomy samples due to the low sample volume of biopsies in the department of pathology which may limit our study. Recent integrative genomic profiling has identified the RB, PI3K and RAS/RAF as the most commonly altered pathways in primary and metastatic PC (Taylor et al., 2010). 

In conclusion, in the present study, we analyzed exclusively 79 consecutive patients of Jordanian origin with prostate cancer mainly at advanced stages and we reported *TP53* mutations in 11.9 % of the patients and absence of *SPOP* and *PIK3CA* mutations in the study population. It is possible that more precise results could be obtained from a study with a larger sample size. Thus, further studies with larger sample size are necessary to compare the site-specific frequencies and define any potential role of these mutations in the development of prostate cancer. Therefore, according to our findings, it is unlikely that in Jordanian population *SPOP* and *PIK3CA* hotspot mutations contribute to prostate cancer or they do but in a much lower percentage. Other mechanisms of *PIK3CA* activation or mutations of other molecular pathways could be involved in the pathogenesis of the disease.
